# Sociodemographic Factors on Contraceptive Use among Ever-Married Women of Reproductive Age: Evidence from Three Demographic and Health Surveys in Bangladesh

**DOI:** 10.3390/medsci5040031

**Published:** 2017-12-06

**Authors:** Iqramul Haq, Saifullah Sakib, Ashis Talukder

**Affiliations:** 1 Department of Statistics, Jagannath University, Dhaka 1100, Bangladesh; shimuljnu107046@gmail.com (I.H.); sakib.du69@gmail.com (S.S.); 2 Statistics Discipline, Khulna University, Khulna 9208, Bangladesh

**Keywords:** family planning, contraceptive use, Demographic and Health Survey, ever-married women, sociodemographic factors, logistic regression

## Abstract

Contraceptive use among married women of reproductive age has showed a substantial progress over the last few decades in Bangladesh. This study explores the sociodemographic factors associated with contraceptive use among ever-married women of reproductive age in Bangladesh by utilizing the information extracted from three of the Bangladesh Demographic and Health Surveys (BDHSs) in 1993–1994, 2004 and 2014. Bivariate analysis was conducted by performing chi-squared test of independence to explore the relationship between selected sociodemographic factors and dependent variables. To know the adjusted effects of covariates, a popular binary logistic regression model was considered. Respondents’ current age, place residence, division religion, education, age at first marriage, family planning (FP) media exposure, ideal number of children and fertility preferences are the significant determinants according to the most recent survey, BDHS 2014. However, wealth index and a respondent’s current working status were found to be significant factors only in BDHS 2004. The results of the study strongly recommend efforts to increase the education level among poor people, particularly among women in Bangladesh. Program interventions, including health behavior education and family planning services and counselling, are especially needed for some categories of the population, including those living in rural areas, Sylhet, Chittagong and Dhaka divisions, as well as illiterate and Muslim ever-married women.

## 1. Introduction

A high population growth rate is one of the leading social problems experienced by the developing world. It is responsible not only for increasing the level of poverty but also for decreasing life expectancy [1,2,3]. Contraception proves to be an effective medical intervention for controlling the fertility rate and also very helpful in developing maternal and child health [4].

Over the last 20 years, contraceptive use has shown a remarkable increase in all over the world, particularly in developing countries. The growing use of contraceptive methods in developing countries has reduced unintended pregnancies and maternal mortality by 40% [5]. Moreover, a study conducted in Bangladesh [6] provides evidence that the unintended pregnancy rate was higher (33%) among women who used contraceptives before their last pregnancy than women (23%) who did not use any contraceptives. 

According to the reaffirmed commitments of the United Nations General Assembly, the new sustainable development agenda includes two targets (Target 3.7 and Target 5.6) relevant for family planning [7]. Target 3.7 states that, “by 2030, ensure universal access to sexual and reproductive health-care services, including for family planning, information and education, and the integration of reproductive health into national strategies and programmes”. On the other hand, the statement of Target 5.6 is to “ensure universal access to sexual and reproductive health and reproductive rights as agreed in accordance with the Programme of Action of the International Conference on Population and Development and the Beijing Platform for Action and the outcome documents of their review conferences”.

In the mid-1970s, the total fertility rate (TFR) of Bangladesh was more than six births per woman and the contraceptive prevalence rate (CPR) was less than 10% [8]. According to the 1993–1994 Bangladesh Demographic and Health Survey (BDHS), the CPR had risen to 45% and besides the TFR had fallen to 3.4 [9]. The 2014 BDHS showed that 54% of currently married women of reproductive age in Bangladesh are using modern contraception methods, and the TFR had fallen to 2.3 [10]. 

Despite the improvement of declining TFR in Bangladesh, women of reproductive age of this country need to pay special attention to contraceptive use, since Bangladesh still has a long way to go to reach the targeted CPR level of over 70% [11]. To increase CPR, an explainable and evidence-based strategy is needed. In this context, identification of possible factors for contraceptives use is one of the major component to formulate this strategy.

In the past, several studies have been conducted in Bangladesh to identify the factors that have significant impact on contraceptive use [12,13,14,15,16]. Most of this research was based on the nationally representative sample surveys. This indicates a comparative study can be performed to identify the factors having significant effects on contraceptive use in Bangladesh by using national surveys conducted in different years. Therefore, the main objective of our study is to determine the major sociodemographic determinants of contraceptive use in Bangladesh, using the data extracted from three of the BDHSs conducted in 1993–1994, 2004 and 2014.

## 2. Materials and Methods 

### 2.1. Data Sources

The analysis is based on secondary data obtained from the 1993–1994, 2004 and 2014 BDHSs (data freely available at: http://dhsprogram.com/data/available-datasets.cfm). The samples for 1993–1994 to 2014 BDHSs were nationally representative and cover the entire population residing in noninstitutionalized dwelling units in the country. The survey used the sampling frame from the list of enumeration areas (EAs) from the 1991, 2001 and 2011 Population and Housing Censuses of the People’s Republic of Bangladesh, provided by Bangladesh Bureau of Statistics (BBS) [10].

The BDHSs covered 9681, 10,811 and 17,989 residential households from 1993–1994, 2004 and 2014, respectively. From these sampled households, 9640, 11,440 and 17,886 ever-married women were interviewed, respectively. In this study, data are restricted to ever-married women aged 15–49. Based on these criteria, sample sizes for this study from the several BDHSs were 9495 ever-married women in 1993–1994, 11,290 ever-married women in 2004 and 17,863 ever-married women in 2014. Data were weighted to represent the structure of the Bangladeshi population using weighting factors provided with the BDHS [9,10,17].

### 2.2. Dependent Variable

The dependent variable was current contraceptive use, categorized dichotomously as a “Yes/No” variable. Respondents who were currently using a contraceptive method were categorized as “Yes”, otherwise as “No”.

### 2.3. Independent Variables 

Besides the dependent variable, we also considered a respondent’s current age (categorized into seven categories), place residence (rural, urban), division (Barisal, Chittagong, Dhaka, Khulna, Rajshahi, Rangpur and Sylhet), religion (Islam, others), wealth index (poor, middle, rich), level of education (no education, primary, above secondary), respondent’s current working status (not working, working outside), respondent’s age at first marriage (<18 years, ≥18), family planning (FP) media exposure (no, yes), ideal number of children (0–1, 2–3, 4+) and fertility preferences (no more, have another, undecided, declared infecund) as potential factors for contraception practice in Bangladesh.

### 2.4. Statistical Methods

Frequency distribution was used to describe the background characteristics of the respondents. Two-way contingency tables along with Pearson’s chi-squared test were used to examine the relationships between sociodemographic factors and a dependent variable. To get the adjusted effects of selected factors, we consider a statistical model appropriate for binary response, namely binary logistic regression [18] model in multivariate setup. The result was presented as odds ratio (OR) and 95% confidence interval (CI). The Statistical Package for Social Science (SPSS v20.0, IBM Corporation, Armonk, New York, NY, USA) software was used for data analysis.

## 3. Results

The percentage of women in each category of the selected variables for each survey point are displayed in Table 1. With regard to the respondent’s current age, about half of the women in all three surveys were under age 30. About 7% respondents in all surveys were residing in Barisal division, while slightly over one-third of the respondents live in Dhaka division. Considering the educational status, in the first two surveys, about half of the respondents (58.1% in 1993–1994 and 41.2% in 2004) had no education, while in BDHS 2014, this rate was only one-quarter (24.9%). With respect to the highest educational level, there is a clear indication that the percentage of women attaining secondary and higher education has been increasing in Bangladesh since 1993–1994. The percentage of the respondents who resides in urban areas have increased from 11.5% in 1993–1994 to 28.3% in 2014. Across the three BDHSs, the proportion of Muslims showed a slight increase from 87.8% to 90.1% between 1993–1994 and 2014. The proportion of women with exposure to family planning information in the media declined from 45.5% in 1993–1994 to 19.9% in 2014. Regarding age at first marriage of the respondents, the percentage of marriage took place before 18 years of age has declined from 88.1% in 1993–1994 to 76.5% in 2014 BDHS. In terms of household wealth quintiles, the proportion of women in middle income households has increased since 1993–1994. The proportion of respondent’s working status rose from 16% in 1993–1994 to 33.1% in 2014. The percentage of women reporting three as an ideal family size have increased in recent times. Regarding women’s fertility preferences, the percentage of women wanting no more children has increased slightly from 48.4% in 1993–1994 to 56.7% in 2014. Concerning contraception use, the percentage of women practicing contraception increased from 42% in 1993–1994 to 58.9% in 2014.

The prevalence of contraceptive use at different categories is examined and results are displayed in Table 2. In all the three survey years, the factors significantly associated (*p <* 0.001 or *p <* 0.05) with contraceptive use are respondent’s current age, division, place of residence, religion, level of education, respondent’s age at first marriage, FP media exposure, ideal number of children and fertility preferences. Wealth index is found significant (*p <* 0.001) in BDHS 1993–1994 and 2004 data. However, the selected covariate ‘respondent’s current working status’ is reported as a significant factor (*p <* 0.001) in the 1993–1994 and 2014 BDHSs. 

To assess the adjusted effects of the selected explanatory variables on contraceptive use in each survey point, we considered three logistic regression models. The results are presented in Table 3. The regression analysis revealed that in 1993–1994, women aged 20–24 years were 60% (OR = 1.60, 95% CI = (1.35, 1.90)) more likely to use contraception than the women aged 15–19 years. In 2004 and 2014, the trend also starts with women aged 20–24 years. According to BDHS 1993–1994, women aged 45–49 were only 3% (OR = 1.03, 95% CI = (1.07, 1.63)) more likely to use contraceptives than women aged 15–19 years, while in 2014, the odds were 64% (OR = 0.36, 95% CI = (0.30, 0.42)) less likely. In 1993–1994, the women from Chittagong division (OR = 0.55, 95% CI = (0.45, 0.67)) were less likely and those of Khulna (OR = 1.61, 95% CI = (1.30, 1.99)) and Rajshahi (OR = 1.60, 95% CI = (1.31, 1.96)) divisions were more likely to use contraceptives compared to those of Barisal division. Besides in 2014, the women from Sylhet (OR = 0.57, 95% CI = (0.48, 0.68)), Chittagong (OR = 0.72, 95% CI = (0.62, 0.84)) division were less likely and Khulna (OR = 1.20, 95% CI = (1.02, 1.41)), Rajshahi (OR = 1.37,95% CI = (1.17, 1.60)) and Rangpur (OR = 1.40, 95% CI = (1.20, 1.65)) divisions were more likely to use contraceptives compared to those of Barisal division. This indicated that, over time, contraceptive use rates are higher in all divisions except Chittagong and Sylhet divisions.

With regards to education in all three surveys period, women who attained primary education and again those having secondary and higher levels of education were more likely to use contraceptives than women having no education. In 2014, the rural women were 16% (OR = 0.84, 95% CI = (0.77, 0.91)) less likely to prefer contraceptives than their urban counterparts. For religion in all three surveys, women belonging to the non-Muslim religious groups had more likely used contraceptives compared with Muslim women.

In 1993–1994, the odds of women who were exposed to family planning information in the media were 16% (OR = 1.16, 95% CI = (1.02, 1.31)) more likely to use modern contraception as compared to married women that were not exposed family planning information in the media. Among the wealth index, the results showed that for the 2004 BDHS, women from middle and rich households was (12% and 19%, respectively) more likely to use contraceptives than women in the poor households. Moreover, for the last two surveys (BDHSs 2004 and 2014), the women who thought that four or more as their ideal number of children were (37% and 27%, respectively) less likely to use modern contraception than women whose ideal number of children was 0 or 1. In all three surveys, women who wanted to have another child and as well as women who were uncertain about having another child were less likely to use modern contraception than women who wanted no more children.

## 4. Discussion and Conclusions

Our study was designed to identify the major factors contributing to the changes in contraceptive use in the last two decades, by using the information extracted from the 1993–1994, 2004, and 2014 BDHSs.

The findings of the study provide evidence that contraceptive use decreases with age, indicating older women were more reluctant to use contraceptives than their younger counterparts. This type of relationship between age and contraceptive use has been observed similar to another existing study [19]. Consistent increase of contraceptive use rate from age of 25 to 44 years indicated that in the reproductive period, the better part of women reached their desired number of children and then were willing to terminate a pregnancy by using modern contraceptive methods [20].

Division also mattered in the pattern of contraceptive use. Our analysis strongly indicated that, over time, contraceptive use rates are higher in all divisions except Chittagong and Sylhet divisions. Previous studies also showed the conservativeness of the residents of Sylhet and Chittagong [21,22]. One of the possible reasons for this result is that the lack of concentration of policy makers on certain divisions in Bangladesh. Therefore, we strongly recommend that policymakers implement more programs associated with family planning in every division of Bangladesh. 

The results of the study confirmed that women who attained primary education and again those with secondary and higher levels of education were more likely to use contraceptives than women with no education. This is because higher educational attainment can provide opportunity for better information on contraceptive methods and better access to services. Education can generate an openness among women to new ideas, such as family planning methods [23]. Moreover, women with more education are more likely to be engaged in professional and other employment activities, and thus may be more likely to want to limit their number of children. Several authors have also found a positive relationship between female education and contraceptive use in Bangladesh [22,23,24]. Our findings are consistent with most of the literature of South Asia and elsewhere [20,25,26,27,28,29,30]. Women with more education were more likely to use contraceptives than uneducated women in India as well as Bangladesh [31].

According to previous studies, contraceptive use rates are expected to be higher in urban areas [20,22,23,31]. Our analysis also suggested that urban women were more likely to use contraception than rural women. This may be due to have better access to contraception of urban women than rural women. Urban women are usually keen to accept any method of contraception [32].

Religion has been detected to play an important role in using contraception. With regards to religion in all three surveys, Muslim women have lower odds of contraceptive use compared with non-Muslim women. This indicates that the less favorable attitude of Muslims toward family planning is gradually changing. Other studies also observed similar patterns [23,33]. Muslim women are likely to have a lower approval rate for contraceptive use [32].

Our analysis reveals a significant association between contraceptive practice and women with exposure to family planning information in the media. As for exposure to family planning messages via various forms of mass media, the results were significant in all three surveys. This finding is consistent with those earlier conducted in Bangladesh [22,34]. This finding is similar to that found by a study conducted in South Asia [20,35]. Of the respondents, those women who had no exposure to mass media were less likely to use modern methods than those who did have exposure to media in India [31]. Mass media exposure (radio/TV) has an important effect on reproductive behavior [32]. Contraceptive use and notions of ideal family size were changing at the same time as the use contraceptives [35]. One of the explanations for this result is that media can both apprize and motivate couples, even about such complex subjects as their reproductive means and goals.

In all three survey periods, age at first marriage was also found a significant factor. However, we did not find too much progress regarding to child marriage. Therefore, we strongly suggest the policy makers to take initiatives for reducing child marriage and increasing the rate of contraceptive use among married women in Bangladesh. 

In terms of household wealth quintiles, the results showed that women from middle and rich households were more likely to use contraceptives than women in the poor quintile in 2004. A similar finding is observed in studies conducted in different African countries [36,37]. However, no significant effect of wealth quintiles was observed in other two survey periods.

Regarding ideal family size, the trend showed that for the last two surveys, only women who reported four or more as their ideal number of children were less likely to use contraception than women whose ideal number of children was 0 or 1 in 2004 and 2014. The relationship between ideal family size and contraceptive use has been found to be significant, which corresponds with many of the previous studies [19,38].

Women’s fertility preference is also an important determinant of contraceptive use. In all three surveys, women who wanted to have another child and as well as women who were uncertain about having another child were less likely to use contraception than women who wanted no more children. This finding is similar to the earlier study [39]. One possible explanation for this could be that women using contraceptives have more control over their reproductive health [40].

In summary, we conclude that a respondent’s current age, place residence, division, religion, level of education, age at first marriage, FP media exposure, ideal number of children and fertility preferences are the significant determinants according to the most recent survey, BDHS 2014. However, wealth index and a respondent’s current working status were found to be significant factors only in BDHS 2004. The results of the study strongly recommend efforts to increase the education level among poor people, particularly within the women in Bangladesh. Program interventions, including health behavior education and family planning services and counselling, are especially needed for some categories of the population, including those living in rural areas, Sylhet, Chittagong and Dhaka divisions, illiterate women, and Muslim ever-married women. According to the FP 2020 of Bangladesh, the Sylhet and Chittagong divisions demand special focus to increase the use of contraceptives to 60% by 2021. This will be necessary as we move forward to address the goals of sustainable development goals.

## Figures and Tables

**Table 1:** Percentage distribution of characteristics of the respondents, 1993–1994, 2004 and 2014 Bangladesh Demographic and Health Surveys (BDHSs).

Characteristics	BDHS 1993–1994		BDHS 2004		BDHS 2014	
	No. of Women	(%)	No. of Women	(%)	No. of Women	(%)
**Age group**						
15–19	1271	13.4	1598	14.2	2029	11.4
20–24	2033	21.4	2202	19.5	3224	18.0
25–29	2012	21.2	2013	17.8	3390	19.0
30–34	1456	15.3	1793	15.9	3047	17.1
35–39	1197	12.6	1457	12.9	2315	13.0
40–44	871	9.2	1160	10.3	2092	11.7
45–49	655	6.9	1066	9.4	1766	9.9
**Division**						
Barisal	600	6.3	710	6.3	1111	6.3
Chittagong	2503	26.4	2023	17.9	3301	17.9
Dhaka	2907	30.6	3521	31.2	6223	31.2
Khulna	1203	12.7	1373	12.2	1838	12.2
Rajshahi	2281	24.0	2946	26.1	2103	26.1
Rangpur	NA	NA	NA	NA	2056	NA
Sylhet	NA	NA	717	6.4	1232	6.4
**Education level**						
No education	5529	58.2	4694	41.6	4455	24.9
Primary	2542	26.8	3300	29.2	5209	29.2
Above secondary	1425	15.0	3296	29.2	8199	45.9
**Place of residence**						
Urban	1096	11.5	2551	22.6	5047	28.3
Rural	8399	88.5	8740	77.4	12,816	71.7
**Religion**						
Islam	8332	87.8	10,165	90.0	16,096	90.1
Others	1163	12.2	1126	10.0	1767	9.9
**FP media exposure**						
No	5175	54.5	6469	57.3	14,316	80.1
Yes	4320	45.5	4822	42.7	3547	19.9
**Age at first marriage**						
<18	8349	87.9	9739	86.3	13,657	76.5
18+	1146	12.1	1552	13.7	4206	23.5
**Wealth index**						
Poor	3821	40.2	4499	39.8	6767	37.9
Middle	1570	16.5	4564	19.7	3560	19.9
Rich	4103	43.2	2228	40.4	7536	42.2
**Current working status**						
No	7971	84.0	8747	77.5	11,947	66.9
Yes	1517	16.0	2543	22.5	5912	33.1
**Ideal No. of children**						
0–1	156	1.6	288	2.6	1127	6.3
2–3	7265	76.5	9425	83.5	12,921	85.7
4+	2074	21.8	1577	14.0	3816	8.0
**Fertility preference**						
No more	4292	48.4	5660	50.1	9555	56.7
Have another	3227	36.4	3561	31.6	5293	31.4
Undecided	209	2.4	217	1.9	462	2.7
Declared infecund	282	3.2	379	3.4	561	3.3
**Contraception use status**						
Not using	5481	57.7	5140	45.5	7336	41.1
Using	4014	42.3	6152	54.5	10,527	58.9

FP: Family Planning; NA: not available; No.: Number.

**Table 2:** Percentage distribution of use of modern contraception among ever-married women age 15–49, by selected background characteristics, BDHSs 1993–1994, 2004 and 2014.

Characteristics	BDHS 1993–1994		BDHS 2004		BDHS 2014	
	Contraception Using Status		Contraception Using Status		Contraception Using Status	
	Yes (%)	No (%)	Yes (%)	No (%)	Yes (%)	No (%)
**Age group**						
15–19	23.8	76.2	40.6	59.4	50.0	50.0
20–24	36.4	63.6	51.0	49.0	58.1	41.9
25–29	48.3	51.7	58.9	41.1	64.9	35.1
30–34	53.5	46.5	64.5	35.5	70.6	29.4
35–39	53.8	46.2	65.3	34.7	67.8	32.2
40–44	46.8	53.2	56.0	44.0	54.5	45.5
45–49	25.7	74.3	40.9	59.1	32.6	67.4
	*p* < 0.001		*p* < 0.001		*p* < 0.001	
**Division**						
Barisal	45.3	54.7	51.5	48.5	59.9	40.1
Chittagong	27.4	72.6	43.9	56.1	52.0	48.0
Dhaka	42.0	58.2	55.8	44.2	59.2	40.8
Khulna	53.0	57.0	60.0	40.0	63.1	36.9
Rajshahi	52.5	47.5	64.6	38.4	66.2	33.8
Rangpur	NA	NA	NA	NA	66.1	33.9
Sylhet	NA	NA	28.4	71.6	44.5	55.5
	*p* < 0.001		*p* < 0.001		*p* < 0.001	
**Education level**						
No education	38.1	61.9	53.2	46.8	54.5	45.5
Primary	44.4	55.6	54.5	45.5	59.6	40.4
Above secondary	54.7	45.3	56.2	43.8	60.9	39.1
	*p* < 0.001		*p* < 0.05		*p* < 0.001	
**Place of residence**						
Urban	50.9	49.1	58.5	41.5	61.5	38.5
Rural	41.1	58.9	53.3	46.7	57.9	42.1
	*p* < 0.001		*p* < 0.001		*p* < 0.001	
**Religion**						
Islam	41.1	58.9	53.7	46.3	58.2	41.8
Others	50.9	49.1	61.8	38.2	65.6	34.4
	*p* < 0.001		*p* < 0.001		*p* < 0.001	
**FP media exposure**						
No	38.6	61.4	51.6	48.4	58.2	41.8
Yes	46.7	53.3	58.3	41.7	61.7	38.3
	*p* < 0.001		*p* < 0.001		*p* < 0.001	
**Age at first marriage**						
<18	42.9	57.1	55.0	45.0	59.5	40.5
18+	37.9	62.1	51.3	48.7	57.0	43.0
	*p* < 0.01		*p* < 0.01		*p* < 0.01	
**Wealth index**						
Poor	38.9	61.1	51.6	48.4	58.7	41.3
Middle	42.4	57.6	54.7	45.3	59.7	40.3
Rich	45.4	54.6	57.2	42.8	58.7	41.3
	*p* < 0.001		*p* < 0.001		*p* = 0.546	
**Current working status**						
No	41.3	58.7	54.2	45.8	57.5	42.5
Yes	47.3	52.7	55.6	44.4	61.9	38.1
	*p < 0.001*		*p = 0.211*		*p < 0.001*	
**Ideal No. of children**						
0–1	48.1	51.9	61.6	38.4	64.3	35.7
2–3	46.2	53.8	56.8	43.2	59.8	40.2
4+	28.2	71.8	39.1	60.9	45.5	54.5
	*p* < 0.001		*p* < 0.001		*p* < 0.001	
**Fertility preference**						
No more	50.8	49.2	67.3	32.7	68.3	31.7
Have another	28.6	71.4	44.6	55.4	51.8	48.2
Undecided	16.7	83.3	40.1	59.9	52.8	47.2
Declared infecund	2.5	97.5	0.5	99.5	6.1	93.9
	*p* < 0.001		*p* < 0.001		*p* < 0.001	

**Table 3:** Odds ratios (OR) with 95% confidence intervals (CI) of explanatory variables for the use of modern contraception among ever-married women in Bangladesh, in 1993–1994, 2004 and 2014 obtained from logistic regression model.

Characteristics	BDHS 1993–1994		BDHS 2004		BDHS 2014	
	OR	95% CI Lower-Upper	OR	95% CI Lower-Upper	OR	95% CI Lower-Upper
**Age group**						
15–19 (ref)	1.00		1.00		1.00	
20–24	1.60 ***	1.35–1.90	1.52 ***	1.32–1.75	1.25 ***	1.12–1.41
25–29	2.38 ***	1.99–2.85	1.97 ***	1.69–2.30	1.39 ***	1.22–1.57
30–34	2.74 ***	2.25–3.33	2.48 ***	2.09–2.93	1.66 ***	1.45–1.92
35–39	2.86 ***	2.32–3.51	2.51 ***	2.09–3.01	1.44 ***	1.24–1.68
40–44	2.38 ***	1.90–2.98	1.91 ***	1.57–2.32	0.85 ***	0.73–0.99
45–49	1.03 ***	1.07–1.63	1.28 ***	1.05–1.57	0.36 ***	0.30–0.42
**Division**						
Barisal (ref)	1.00		1.00		1.00	
Chittagong	0.55 ***	0.45–0.67	0.73 **	0.61–0.87	0.72 ***	0.62–0.84
Dhaka	0.99	0.82–1.21	1.17	0.98–1.39	1.01	0.88–1.17
Khulna	1.61 ***	1.30–1.99	1.36 **	1.12–1.65	1.20 *	1.02–1.41
Rajshahi	1.60 ***	1.31–1.96	1.82 ***	1.53–2.18	1.37 ***	1.17–1.60
Rangpur	NA	NA	NA	NA	1.40 ***	1.20–1.65
Sylhet	NA	NA	0.38 ***	0.30–0.49	0.57 ***	0.48–0.68
**Education level**						
No education (ref)	1.00		1.00		1.00	
Primary	1.26 ***	1.13–1.41	1.12 *	1.01–1.24	1.16 **	1.06–1.26
Above secondary	2.17 ***	1.87–2.53	1.34 ***	1.19–1.50	1.23 ***	1.11–1.35
**Place of residence**						
Urban (ref)	1.00		1.00		1.00	
Rural	0.87	0.75–1.01	0.87 **	0.78–0.96	0.84 ***	0.77–0.91
**Religion**						
Islam(ref)	1.00		1.00		1.00	
Others	1.40 ***	1.21–1.60	1.56 ***	1.36–1.80	1.36 ***	1.21–1.52
**FP media exposure**						
No (ref)	1.00		1.00		1.00	
Yes	1.16 **	1.02–1.31	1.20 ***	1.10–1.32	1.10 *	1.01–1.20
**Age at first marriage**						
<18 (ref)	1.00		1.00		1.00	
18+	0.70 ***	0.61–0.82	0.79 ***	0.70–0.89	0.89 **	0.83–0.97
**Wealth index**						
Poor (ref)	1.00		1.00		1.00	
Middle	1.09	0.96–1.25	1.12 *	1.02–1.26	1.09	0.99–1.19
Rich	1.02	0.90–1.15	1.19 **	1.06–1.32	1.01	0.92–1.10
**Current working status**						
No (ref)	1.00		1.00		1.00	
Yes	0.99	0.87–1.11	0.82 ***	0.75–0.91	1.07	0.90–1.14
**Ideal No. of children**						
0–1 (ref)	1.00		1.00		1.00	
2–3	1.31	0.93–1.85	0.96	0.74–1.24	0.98	0.86–1.12
4+	0.81	0.56–1.16	0.63 **	0.48–0.84	0.77 **	0.65–0.92
**Fertility preference**						
No more (ref)	1.00		1.00		1.00	
Have another	0.45 ***	0.40–0.51	0.58 ***	0.52–0.65	0.50 ***	0.46–0.55
Undecided	0.26 ***	0.18–0.38	0.48 ***	0.36–0.64	0.49 ***	0.41–0.60
Declared infecund	0.03 ***	0.02–0.07	0.03 ***	0.01–0.06	0.05 ***	0.03–0.07

ref: reference group; Statistical significance: * *p* < 0.05, ** *p* <0.01, *** *p* < 0.001.

## References

[1] Mondal M.N.I., Ullah M.M., Islam M.R., Rahman M.S., Khan M.N., Ahmed K.M., Islam M.S. (2015). Sociodemographic and Health Determinants of Inequalities in Life Expectancy in Least Developed Countries. Int. J. MCH AIDS.

[2] Mondal M.N.I., Shitan M. (2013). Impact of Socio-Health Factors on Life Expectancy in the Low and Lower Middle Income Countries. Iran. J. Public Health.

[3] Mondal M.N.I., Shitan M. (2014). Relative Importance of Demographic, Socioeconomic and Health on Life Expectancy in the Low- and Lower-Middle-Income Countries. J. Epidemiol..

[4] Oye-Adeniran B.A., Adewole I.F., Umoh A.V., Oladokun A., Ghadegesin A., Ekanem E.E. (2006). Community-based Study of Contraceptive Behavior in Nigeria. Afr. J. Reprod. Health.

[5] Cleland J., Agustin C.A., Peterson H., Ross J., Tsui A. (2012). Contraception and health. Lancet.

[6] Noor F.R., Rahman M.M., Rob U., Bellows B. (2012). Unintended pregnancy among rural women in Bangladesh. Int. Q. Community Health Educ..

[7] United Nations A/RES/70/1—Transforming Our World: The 2030 Agenda for Sustainable Development. https://sustainabledevelopment.un.org/post2015/transformingourworld.

[8] Islam M.N., Islam M.M. (1993). Biological and behavioural determinants of fertility in Bangladesh: 1975–1989. Asia-Pac. Popul. J..

[9] National Institute of Population Research and Training (NIPORT) (1994). Bangladesh Demographic and Health Survey 1993–1994.

[10] National Institute of Population Research and Training (NIPORT) (2016). Bangladesh Demographic and Health Survey 2014.

[11] National Institute of Population Research and Training, Mitra and Associates (2012). Bangladesh Demographic and Health Survey, 2011.

[12] Kibria G.M.A., Hossen S., Barsha R.A.A., Sharmeen A., Paul S.K. (2016). Factors affecting contraceptive use among married women of reproductive age in Bangladesh. J. Mol. Stud. Med. Res..

[13] Kamal S.M., Islam M.A. (2010). Contraceptive use: Socioeconomic correlates and method choices in rural Bangladesh. Asia Pac. J. Public Health.

[14] Kamal S.M. (2015). Socioeconomic factors associated with contraceptive use and method choice in urban slums of Bangladesh. Asia Pac. J. Public Health.

[15] Laskar M.S., Mahbub M.H., Yokoyama K., Inoue M., Harada N. (2006). Factors associated with contraceptive practices of married women in Bangladesh with respect to their employment status. Eur. J. Contracept. Reprod. Health Care.

[16] Mannan H.R. (2002). Factors in contraceptive method choice in Bangladesh: Goals, competence, evaluation and access. Contraception.

[17] National Institute of Population Research and Training (NIPORT) (2005). Mitra and Associates, and Macro International Inc. Bangladesh Demographic and Health Survey 2004.

[18] Hosmer D.W., Lemeshow S. (2000). Applied Logistic Regression.

[19] Khan H., Raeside R. (1997). Factors affecting the most recent fertility rates in urban-rural Bangladesh. Soc. Sci. Med..

[20] Osmani A.K., Reyer J.A., Osmani A.R., Hamajima N. (2015). Factors influencing contraceptive use among women in Afghanistan: Secondary analysis of Afghanistan Health Survey 2012. Nagoya J. Med. Sci..

[21] Mahmud M., Islam M.M. (1995). Adolescent Contraceptive Use and Its Determinants in Bangladesh: Evidence from Bangladesh Fertility Survey 1989. Contraception.

[22] Kabir A. (2001). Determinants of the Current Use of Contraceptive Methods in Bangladesh. J. Med. Sci..

[23] Masuda Mohsena M., Kamal M. (2014). Determinants of Contraceptive Use in Bangladesh. Ibrahim Med. Coll. J..

[24] Ullah M.S., Chakraborty N. (1993). Factors affecting the Use of Contraception in Bangladesh: A Multivariate analysis. Asia Pac. Popul. J..

[25] Saleem S., Bobak M. (2005). Women’s autonomy, education and contraception use in Pakistan: A national study. BMC Reprod. Health.

[26] Al Riyami A., Afifi M., Mabry R.M. (2004). Women’s autonomy, education and employment in Oman and their influence on contraceptive use. Reprod. Health Matters.

[27] Mahmood N., Ringheim K. (1996). Factors affecting contraceptive use in Pakistan. Pak. Dev. Rev..

[28] Saleem A., Pasha G.R. (2008). Women’s reproductive autonomy and barriers to contraceptive use in Pakistan. Eur. J. Contracept. Reprod. Health Care.

[29] Arokiasamy P. (2002). Gender preference, contraceptive use and fertility in India: Regional and development influences. Int. J. Popul. Geogr..

[30] Khan M.A., Khanum P.A. (2000). Influence of son preference on contraceptive use in Bangladesh. Asia-Pac. Popul. J..

[31] Islam M.K. (2017). Contraceptive Use, Method Choice and Discontinuation of Contraception in South Asia. Am. J. Sociol. Res..

[32] Hussain N. (2011). Demographic, Socio-Economic and Cultural Factors Affecting Knowledge and Use of Contraception Differentials in Malda District, West Bengal. J. Community Med. Health Educ..

[33] Kamal N., Mohsena M. (2011). Twenty Years of Field Visits by Family Planning Workers in Bangladesh: Are They Still Needed?. Indian J. Fam. Welf..

[34] Kamal S.M.M. Contraceptive Use and Method Choice in Urban Slum of Bangladesh. Proceedings of the International Conference on Family Planning: Research and Best Practices.

[35] Retherford R.D., Mishra V. (1997). Media exposure increases contraceptive use. Natl. Fam. Health Surv. Bull..

[36] Adebowale S.A., Adedini S.A., Ibisomi L.D., Palamuleni M.E. (2014). Differential effect of wealth quintile on modern contraceptive use and fertility: Evidence from Malawian women. BMC Women’s Health.

[37] Creanga A.A., Gillespie D., Karklins S., Tsui A.O. (2011). Low use of contraception among poor women in Africa: An equity issue. Bull. World Health Organ..

[38] Kamal N., Sloggett A., Cleland J.C. (1999). Area variations in use of modern contraception inrural Bangladesh: A multilevel analysis. J. Biosoc. Sci..

[39] Worku A.G., Tessema G.A., Zeleke A.A. (2015). Trends of modern contraceptive use among young married women based on the 2000, 2005, and 2011 ethiopian demographic and health surveys: A multivariate decomposition analysis. PLoS ONE.

[40] OlaOlorun F.M., Hindin M.J. (2014). Having a say matters: Influence of decisionmaking power on contraceptive use among nigerian women ages 35–49 years. PLoS ONE.

